# Marked variation in newborn resuscitation practice: A national survey in the UK^☆^

**DOI:** 10.1016/j.resuscitation.2012.01.002

**Published:** 2012-05

**Authors:** Chantelle Mann, Carole Ward, Mark Grubb, Barrie Hayes-Gill, John Crowe, Neil Marlow, Don Sharkey

**Affiliations:** a Division of Academic Child Health, University of Nottingham, Nottingham, UK; b Division of Electrical Systems and Applied Optics, University of Nottingham, UK; c Institute for Women's Health, University College London, UK

**Keywords:** NICU, neonatal intensive care unit, DR, delivery room, ANNP, advanced neonatal nurse practitioner, PEEP, positive end expiratory pressure, CPAP, continuous positive airway pressure, NDAU, Neonatal Data Analysis Unit, NS, not statistically significant, Neonatal resuscitation, Survey, Practice variation

## Abstract

**Background:**

Although international newborn resuscitation guidance has been in force for some time, there are no UK data on current newborn resuscitation practices.

**Objective:**

Establish delivery room (DR) resuscitation practices in the UK, and identify any differences between neonatal intensive care units (NICU), and other local neonatal services.

**Methods:**

We conducted a structured two-stage survey of DR management, among UK neonatal units during 2009–2010 (*n* = 192). Differences between NICU services (tertiary level) and other local neonatal services (non-tertiary) were analysed using Fisher's exact and Student's *t*-tests.

**Results:**

There was an 89% response rate (*n* = 171). More tertiary NICUs institute DR CPAP than non-tertiary units (43% vs. 16%, *P* = 0.0001) though there was no significant difference in frequency of elective intubation and surfactant administration for preterm babies. More tertiary units commence DR resuscitation in air (62% vs. 29%, *P* < 0.0001) and fewer in 100% oxygen (11% vs. 41%, *P* < 0.0001). Resuscitation of preterm babies in particular, commences with air in 56% of tertiary units. Significantly more tertiary units use DR pulse oximeters (58% vs. 29%, *P* < 0.01) and titrate oxygen based on saturations. Almost all services use occlusive wrapping to maintain temperature for preterm infants.

**Conclusions:**

In the UK, there are many areas of good evidence based DR practice. However, there is marked variation in management, including between units of different designation, suggesting a need to review practice to fulfil new resuscitation guidance, which will have training and resource implications.

## 1 Introduction

Whilst the majority of newborn infants successfully transition from fetal life with minimal assistance, up to 10% will need some form of additional support, and 1% will require significant resuscitation.^1^ International clinical guidance describes a standardised approach to newborn resuscitation in the delivery room (DR) and national clinical algorithms are guided by these consensus statements.^2^ These guidelines aim to provide an organised, sequential and standardised approach to DR resuscitation of the newborn.

Though advances in neonatal intensive care have significantly improved outcomes, few large studies have examined consistency of practice in early DR management. The most recent guidance on resuscitation practices and equipment was updated in late 2010 by the International Liaison Committee on Resuscitation (ILCOR), American Heart Association (AHA) and UK Resuscitation Council (UKRC).^2–4^ Since the previous update in 2005, an increasing number of studies into DR management have encouraged evolution of local practice including the avoidance of 100% oxygen for resuscitation to minimise oxidative stress.^5,6^ Further studies suggest use of early CPAP^7^ and pulse oximetry in the DR^8,9^ may be useful, although additional studies are needed to examine outcomes in these areas. Data regarding DR practices from other developed countries have suggested discordance between current evidence and standard clinical practice in recent years.^10,11^ There is clearly potential to develop and improve care during the crucial first minutes of life, with a view to further improving clinical outcomes for term and preterm babies.

Following the establishment of neonatal networks in the UK, lead tertiary centres have a critical role not only in ensuring best practice within their service, but also in fostering this across their regional network. As new evidence emerges on DR resuscitation it is essential that all units delivering and resuscitating newborn babies are equipped to follow best practice. In this respect, data on current UK newborn resuscitation practices are essential in ensuring neonatal outcomes are optimised.

The aims of the present study, therefore, were:

1. To establish DR resuscitation practices for term and preterm babies in the UK.
2. To identify differences in practice between tertiary NICUs, and non-tertiary neonatal services across the UK.

## 2 Methods

We conducted a two stage structured survey of DR management among neonatal services in the UK (see Supplement A). The survey focussed on establishing and comparing DR practice in the domains of ventilatory support, oxygen therapy, assessment and monitoring, and transfer to NICU. The survey focussed on DR practice, thermoregulation, respiratory management and equipment use. With the establishment of UK regional neonatal networks, we compared the services delivering the majority of intensive care (tertiary NICUs), with those providing more limited services (local neonatal units, and Special Care Units) combined together as “non-tertiary” units for the purposes of analysis.

To maximise returns, we utilised telephone, and postal questionnaires. Repeat questionnaires were sent to non-responders. The primary survey was conducted between May and December 2009. Data from the primary survey suggested marked differences in air/oxygen blender availability and initial oxygen delivery. To address this we conducted a supplementary questionnaire between May and December 2010 to establish blender availability and utilisation, in term and preterm infants, within tertiary NICUs. In total 192 hospitals were surveyed, including all tertiary units, and comparisons were drawn between services of differing designation. Unit designation was defined by self reporting and from the 2009 Neonatal Data Analysis Unit (NDAU) database.^12^ Services in Scotland and Northern Ireland were identified separately and their type of activity established. Data were analysed using Fisher's exact test for two-sample comparisons and Student's *t*-test for numerical variables. In all cases *P* < 0.05 was considered statistically significant. This nationwide service evaluation did not require ethical approval.

## 3 Results

Our response rate of 89% (171 services) comprised 65 tertiary NICUs, and 106 non-tertiary units. At the time of the 2010 supplemental survey, 3 NICUs had been reclassified to non-tertiary units (*n* = 62). Respondent designation included consultants (*n* = 43), senior paediatric/neonatal trainees (*n* = 88), junior paediatric/neonatal trainees (*n* = 3), ANNP/senior neonatal nurses (*n* = 26), research officer (*n* = 1) and unknown status (*n* = 10). Non-responders (11%) were randomly distributed across the UK and comprised of 4 NICUs, 12 local neonatal units, 4 Special Care Units, and one service of unknown designation.

### 3.1 DR ventilatory support

**Fig. 1:**
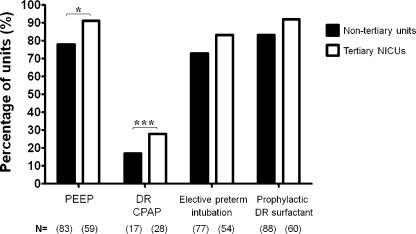
Graph summarising routine ventilatory support provided by UK neonatal units in the delivery room (DR), as percentage of responding units (tertiary NICUs, *n* = 65; non-tertiary local neonatal units and Special Care Units, *n* = 106). Actual numbers of units displayed as *N* = (*x*), *P < 0.05, ***P < 0.001.

The most commonly used ventilation device was the Neopuff (Infant T-Piece Resuscitator, Fisher & Paykel Healthcare), used by 83 (49%) of the responding units. More tertiary units use ventilation devices capable of delivering PEEP, compared to non-tertiary units (*P* = 0.04), or institute early DR CPAP (*P* = 0.0001). Ventilatory support data is summarised in Fig. 1.

There was no significant difference between tertiary and non-tertiary units in terms of elective intubation for preterm infants. The median age below which preterm infants were electively intubated was 28 weeks gestation (range 24–32 weeks) among all units. Among those units implementing elective intubation, 121 (92%) subsequently administered surfactant in the DR, with no significant difference between tertiary and other units. Including all responding units, 92% (60) of tertiary units would administer DR surfactant if a preterm baby were intubated, similar to 84% (88) of non-tertiary units (NS).

### 3.2 Oxygen therapy

**Fig. 2:**
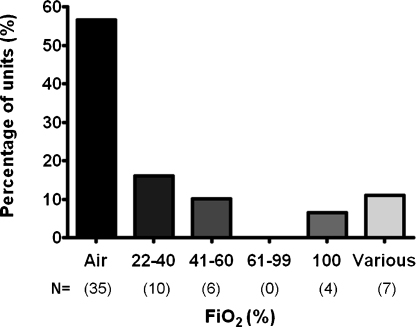
Graph displaying the specific supplemental oxygen concentrations used by 62 tertiary NICUs, when commencing resuscitation for preterm babies. Actual numbers of units displayed as *N* = (*x*). ‘Various’ refers to those services which allow individual practice variation.

There was marked variation in practice around the use of supplemental oxygen during DR resuscitation among tertiary NICU services: 34 (55%) implement a specific local or regional guideline, 8 (13%) follow national Neonatal Life Support (NLS) guidance only and 20 (32%) follow no specific guideline but allow variation in individual practice. Compared with other services, significantly more tertiary units commence DR resuscitation in air (62% vs. 29%, *P* < 0.0001) and fewer commence resuscitation in 100% oxygen (11% vs. 41%, *P* < 0.0001). Titration of oxygen concentration during resuscitation is significantly more common among tertiary units (57% vs. 33%, *P* = 0.003). In the supplementary survey, tertiary NICUs reported specifically on their oxygen therapy guidance for preterm infant resuscitation (Fig. 2). Of the 20 (32%) units using different initial oxygen concentrations for preterm vs. term infants, the median gestational age under which preterm guidance was implemented was 32 weeks (range 28–37 weeks).

With respect to the availability of oxygen blenders, 2 tertiary units (3%) have none, 17 (27.5%) have blenders in some DRs only, and 43 (69.5%) have blenders in all DRs. Of those tertiary units who commenced resuscitation in 100% oxygen, all reported the presence of oxygen blending facilities in all their DRs.

### 3.3 Temperature regulation

Of all services, 165 (97%) use plastic wrapping for preterm infants below a median gestational age of 30 weeks (range 27–34 weeks), or where birth weight was estimated to be less than 1000 g (median; range 1000–1500 g). Thirty two services used chemical warming mattresses in addition to occlusive wrapping at gestational age below 30 weeks (median; range 26–40 weeks) and there was no significant variation between tertiary and non-tertiary units (16% and 20% respectively; *P* = 0.55).

### 3.4 Assessment and monitoring

**Fig. 3:**
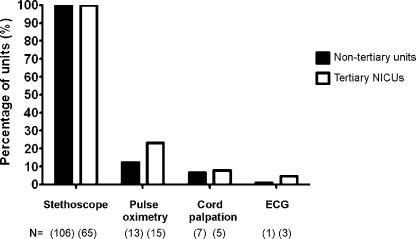
Graph displaying modalities routinely used to assess heart rate in the DR, according to unit designation. Data displayed as percentages of each level service respectively (tertiary NICUs, *n* = 65; non-tertiary local neonatal units and Special Care Units, *n* = 106). Actual numbers of units displayed as *N* = (*x*).

Responding units described a variety of techniques for heart rate assessment in the DR. All units reported routine intermittent praecordial auscultation, whilst many fewer used umbilical cord pulsation, pulse oximetry or ECG during resuscitation (Fig. 3).

Significantly more tertiary units apply pulse oximeters in the DR than non-tertiary units (58% vs. 29%, *P* < 0.01). However, there is no statistical difference in their reported use specifically for HR monitoring during early resuscitation (*P* = 0.13). From the supplementary tertiary unit survey, 36 (58%) units reported that supplemental oxygen was titrated based on pulse oximetry saturation values, but not for all resuscitation scenarios. Eleven (18%) monitor saturations and titrate oxygen specifically for preterm infants, and 4 (7%) units only for prolonged resuscitations. The remainder (*n* = 21) implement oxygen saturation targeting for all infants in whom resuscitation is commenced. The majority of units did not report specific target ranges, but were said to aim for values in the “normal range”.

### 3.5 Transfer to NICU

The use of specialised transport systems, for the transfer of babies from the DR to neonatal unit, varied significantly with 68% of tertiary NICUs and 44% of non-tertiary units using them routinely (*P* = 0.004). A further 5% of tertiary and 8% of non-tertiary units have access to transport systems, which they use in specific circumstances, generally determined by individual clinicians.

There were no statistical differences between units in their typical estimated transfer times from DR to neonatal unit (tertiary units’ median 3 min, range 1–10; non-tertiary units’ median 2 min, range 1–20). For all units, 46 (27%) had a transfer time of ≥5 min and in 53 (31%) units the DR was on a separate floor from the NICU. Significantly more tertiary NICUs routinely utilised monitoring devices during transfer to the neonatal unit (77% vs. 62%, *P* = 0.04). Of those services not attaching monitoring specifically for transfer, 93% of tertiary and 90% of non-tertiary were on the same floor with transfer time <5 min.

## 4 Discussion

These are the first data to describe variation in DR newborn resuscitation practice in the UK, based on responses from 89% of UK neonatal services. As well as demonstrating shared areas of best practice, we have also identified significant variation in DR management which could impact on newborns in both the short and long term. Previously published international data have alluded to a disparity between the current scientific evidence base and clinical uptake into the DR.^13,14^ Importantly, this study demonstrates that, even prior to the revision of the current resuscitation guidelines, many of the new changes, especially in respiratory management and monitoring, were already being implemented by many units.

Neonatal networks were established in the UK in 2004, to ensure better outcomes, particularly for the sickest infants.^15^ Follow-up studies of extremely preterm infants (*n* = 1846), born in England in 2006, identified improved survival for those born in larger more specialised units.^16^ Although some of the potential advantages of networks may take time to filter through to improved outcomes, this can only realistically occur if best practice is implemented promptly as new evidence and guidance becomes available.

It is encouraging to see that the management of preterm infants, arguably those likely to benefit most from good DR practice, shows some convergence in several areas. In this group of infants there is a strong evidence base to support many aspects of early care, including the importance of temperature regulation as highlighted in Project 27/28.^17^ This 2004 UK inquiry into the effect of the quality of neonatal care on preterm survival, documented a 70% increase in the risk of death with an admission temperature to the neonatal unit ≤36.0 °C. During the period of this survey, 97% of all responding centres used occlusive plastic bags/wraps to reduce the risk of hypothermia. This may reflect a progressive uptake of the evidence around the world, since 27% of North American units reported occlusive wrap use in 2004.^11^

The use of elective early intubation in preterm infants <28 weeks, paired with DR surfactant, is also similar across centres. There are some differences in DR ventilation strategies, notably with tertiary units implementing more early CPAP and using ventilation devices capable of delivering PEEP. A number of studies have documented the broad benefits of PEEP in preventing early lung injury,^18,19^ though the evidence supporting use of DR CPAP in preterm infants is more complex. The recent SUPPORT study showed no significant difference in CLD or mortality but a shorter overall ventilation requirement.^20^ Timely appraisal of the developing evidence in this area, and individualised evaluation of our preterm infants, may prompt an evolution in our early ventilatory support practices.

The most striking differences revealed in this study were in the use of air or oxygen for initial resuscitation. Previous data published from Australia and New Zealand in 2004^14^ reported 76% of responding centres commenced resuscitation in 100% oxygen. Guidance at that time recommended this action, though a growing body of evidence already supported the efficacy of commencing in air. Guidance issued since our UK survey was conducted suggests that targeting “normal” oxygen saturation levels is more important than the oxygen concentration used to achieved this, but do not support the use of 100% oxygen where blending facilities are available,^21^ as currently occurs in 7% of tertiary units. When resuscitating preterm infants 56% of tertiary NICUs commence in air, with wide variation in the oxygen concentrations used elsewhere. Lack of clinical consensus may reflect perceived lack of clarity from the literature, and whilst we await the outcomes of randomised trials currently underway, it may be prudent to target our resuscitation to a healthy heart rate, before concluding the optimal oxygen therapy for this group of babies.

The UKRC have recently recommended pulse oximetry wherever resources are available for deliveries with anticipated problems.^4^ They advocate that saturations and heart rate can be reliably obtained after the first 1–2 min from birth. Though we have demonstrated that many more tertiary units have access to pulse oximeters in the DR, their use during resuscitation varies widely from HR monitoring during prolonged resuscitations, to saturation targeting among all preterm infants, despite no previous guidance on appropriate targets. All responding units still use the stethoscope for heart rate assessment but only 16% of units use a pulse oximeter. Among units implementing DR oximetry, several commented on poor reliability during the first minutes after birth and addressing this in the future may increase uptake of technology in the DR. We would agree with the recently revised resuscitation guidelines that research aimed at defining optimal resuscitation practice, especially in the preterm population, is required. Some of the uncertainties around both initial oxygen concentration and targeted saturations in preterm infants are being addressed in 2 current multicentre resuscitation trials.^22^ Until then, it is essential that the resuscitating team are not distracted, either by trying to obtain a reliable signal or continuously adjusting oxygen delivery based on saturations, when ensuring optimal temperature control and an adequate airway.

Heterogeneity in DR practice also has potential implications for medical training. In paediatric training programmes, trainee doctors rotate through a number of general and specialist hospitals to obtain appropriate education and expertise. This includes specific competencies in resuscitation taught by structured resuscitation programmes such as Newborn Life Support (NLS). Our data suggest that trainees may be exposed to wide variation in DR protocols and practices throughout their training, potentially creating confusion and a lack of clarity concerning best practice. Furthermore, this may perpetuate a lack of confidence in implementing newer interventions and therapies in non-tertiary units, with relatively fewer opportunities for practitioners to practice and familiarise themselves with new interventions. Increased standardisation within and between Neonatal networks would go some way to ameliorate this potential problem. The important data gathered during this study will allow individual units to review their practice with similar units across the UK.

As with many national surveys of this nature, there are some limitations. We describe resuscitation practices as reported by the responding practitioner, and though they were each specifically asked to base their responses on local rather than individual practice, these data may not fully represent the actual policies of each unit. In the first instance we categorised neonatal units according to self-reported designation, assigning a level from the NDAU database where this information was not provided by the unit. Nonetheless, we highlight the significant gap that exists between what is thought to be optimal resuscitation practice, i.e. current international and national guidelines, and what was actually occurring prior to their publication.

## 5 Conclusion

We have identified significant variation in DR resuscitation practices among neonatal services in the UK. There are significant differences within specific areas of clinical management despite high quality evidence supporting standardisation. More worrying, there is a suggestion that some practices are not based on current evidence or consensus agreement and this may reflect the lack of good data in some domains. These variations in strategies are consistent with previously published data from other developed countries. The discrepancies between best evidence and current practice are an important target in our endeavours to improve neonatal outcomes and optimise training and practice. As our study was conducted immediately prior to an international update in clinical guidance, it provides a valuable baseline from which to conduct and compare future evaluations. This vital period in newborn care remains understudied and warrants prioritisation when considering research and funding agendas. There is a crucial role for all newborn care services, but especially tertiary NICUs, in appraising current evidence and sharing best practice within their network. By minimising these care differences, we can hope to optimise clinically meaningful neonatal outcomes.

## Conflict of interest statement

The authors declare no financial or other conflicts of interest.

## Financial disclosure

C.W., M.G., B.H.G., J.C., N.M. and D.S. have been supported by funding from Action Medical Research and C.M., M.G., B.H.G., J.C., N.M. and D.S. by a Medical Research Council DPFS portfolio award. N.M. also receives a proportion of funding from the Department of Health's NIHR Biomedical Research Centres funding scheme at UCLH/UCL.
