# Supplementary material for: Marked variation in newborn resuscitation practice: A national survey in the UK
Source: Resuscitation. 2012 May;83(5):607–11. doi: 10.1016/j.resuscitation.2012.01.002 (PMC3350052; doi:10.1016/j.resuscitation.2012.01.002)
Supplement: Supplementary file 1 [file mmc1.doc]

**Supplement A**

**Delivery Room Resuscitation Survey**

(please circle appropriate response)

**1. What device(s) do you use for ventilation in the delivery room?**

Bag & Mask Neopuff T-piece (no PEEP) T-piece (PEEP) Other

**2. Do you electively intubate in the delivery room?**

No Yes At what gestation? Nasal or Oral intubation?

**3. Do you use delivery room surfactant if intubated?**

Yes No

**4. Do you use delivery room CPAP?**

Yes No

**5. What do you assess heart rate with during initial resuscitation?**

Stethoscope Pulse oximetry ECG Other

**6. If you use a pulse oximeter, which one do you use?**

Nelcor Massimo Radical Other

**7. Do you titrate oxygen according to saturations in the delivery room?**

Yes No

**8. Do you start delivery room resuscitation in air or oxygen?**

Air Oxygen **at %**

**9. Do you use plastic bags/wraps in the delivery room?**

No Yes **At what gestation/weight?** Gestation Weight

**10. Do you use Transwarmers in the delivery room?**

No Yes **At what gestation/weight?** Gestation Weight

**11. How do you transfer to the NICU?**

On resuscitaire Transport system Other

**12. Do you use a monitor for transfer?**

No Yes **ECG? Pulse Oximeter?**

**13. How many minutes does it roughly take to go from delivery room to NICU cot?**

minutes

**14. Is your delivery room on the same floor as the NICU?**

Yes No

**Unit: Level: Respondent: Date:**
